# All-Cause Mortality and Serious Cardiovascular Events in People with Hip and Knee Osteoarthritis: A Population Based Cohort Study

**DOI:** 10.1371/journal.pone.0091286

**Published:** 2014-03-07

**Authors:** Gillian A. Hawker, Ruth Croxford, Arlene S. Bierman, Paula J. Harvey, Bheeshma Ravi, Ian Stanaitis, Lorraine L. Lipscombe

**Affiliations:** 1 Women’s College Research Institute, Women’s College Hospital, Toronto, Ontario, Canada; 2 Institute of Health Policy, Management and Evaluation, University of Toronto, Toronto, Ontario, Canada; 3 Institute for Clinical Evaluative Sciences, Toronto, Ontario, Canada; 4 Department of Medicine, University of Toronto, Toronto, Ontario, Canada; 5 Lawrence S. Bloomberg Faculty of Nursing, University of Toronto, Toronto, Ontario, Canada; 6 Division of Orthopaedics, Department of Surgery, University of Toronto, Toronto, Ontario, Canada; Oxford University, United Kingdom

## Abstract

**Background:**

Because individuals with osteoarthritis (OA) avoid physical activities that exacerbate symptoms, potentially increasing risk for cardiovascular disease (CVD) and death, we assessed the relationship between OA disability and these outcomes.

**Methods:**

In a population cohort aged 55+ years with at least moderately severe symptomatic hip and/or knee OA, OA disability (Western Ontario McMaster Universities (WOMAC) OA scores; Health Assessment Questionnaire (HAQ) walking score; use of walking aids) and other covariates were assessed by questionnaire. Survey data were linked to health administrative data to determine the relationship between baseline OA symptom severity to all-cause mortality and occurrence of a composite CVD outcome (acute myocardial infarction, coronary revascularization, heart failure, stroke or transient ischemic attack) over a median follow-up of 13.2 and 9.2 years, respectively.

**Results:**

Of 2156 participants, 1,236 (57.3%) died and 822 (38.1%) experienced a CVD outcome during follow-up. Higher (worse) baseline WOMAC function scores and walking disability were independently associated with a higher all-cause mortality (adjusted hazard ratio, aHR, per 10-point increase in WOMAC function score 1.04, 95% confidence interval, CI 1.01–1.07, p = 0.004; aHR per unit increase in HAQ walking score 1.30, 95% CI 1.22–1.39, p<0.001; and aHR for those using versus not using a walking aid 1.51, 95% CI 1.34–1.70, p<0.001). In survival analysis, censoring on death, risk of our composite CVD outcome was also significantly and independently associated with greater baseline walking disability ((aHR for use of a walking aid  = 1.27, 95% CI 1.10–1.47, p = 0.001; aHR per unit increase in HAQ walking score  = 1.17, 95% CI 1.08–1.27, p<0.001).

**Conclusions:**

Among individuals with hip and/or knee OA, severity of OA disability was associated with a significant increase in all-cause mortality and serious CVD events after controlling for multiple confounders. Research is needed to elucidate modifiable mechanisms.

## Introduction

Globally, aging populations and the growing prevalence of obesity [1], [2] have led to increased population risk for hypertension [3], [4], dyslipidemia [5], diabetes [6], and cardiovascular disease (CVD) [7]. A less well recognized consequence of these trends is the increasing burden of osteoarthritis (OA) [5], [8]. OA is the most common arthritis [9], affecting approximately 10% of the adult population [1]. Symptomatic OA leads to functional limitations, depressed mood and loss of independence [10]. With no cure, OA management focuses on pain relief and preserving physical function [11] using non-pharmacologic and pharmacologic therapies and, ultimately, joint replacement surgery. Despite effective management strategies, OA is under-diagnosed and under-treated. This is in part due to the high co-prevalence of other chronic conditions in people with OA; 90% are estimated to have at least one additional chronic condition [12], with OA and CVD among the most common dyads seen in clinical practice. CVD, in particular, may be perceived as precluding the use of OA therapies (e.g. non-steroidal anti-inflammatory drugs, NSAIDs [13], [14]).

Inadequately treated, people commonly manage OA pain by avoiding activities, like walking, that exacerbate pain [10], [15]. This lack of physical activity may in turn lead to poorer fitness, higher risk of CVD [16], [17], and inadequate self-management of chronic conditions [18]. Nuesch *et al* [19] documented increased all-cause mortality in people with versus without OA in an English population cohort; walking disability predicted increased risk for death. Given the rising numbers with OA, confirmation of these relationships is important. Further, it is important to know whether *among people with hip/knee OA*, severity of OA-related functional limitations predicts adverse outcomes, including serious CVD events and death. Documenting a significant, independent relationship between OA symptom severity and CVD risk would provide evidence for improving OA management to reduce CVD risk and for screening and modifying CVD risk factors in people with OA. In a Canadian population cohort with hip and knee OA, the current study therefore evaluated the prognostic value of baseline levels of hip and knee osteoarthritis (OA) related pain and disability on all-cause mortality and risk for serious CVD events.

## Methods

### Ethics Statement

Participants provided written informed consent to participate in the study. The Women’s College Research Institute Ethics Review Board approved the study.

### Participants

**Figure 1:**
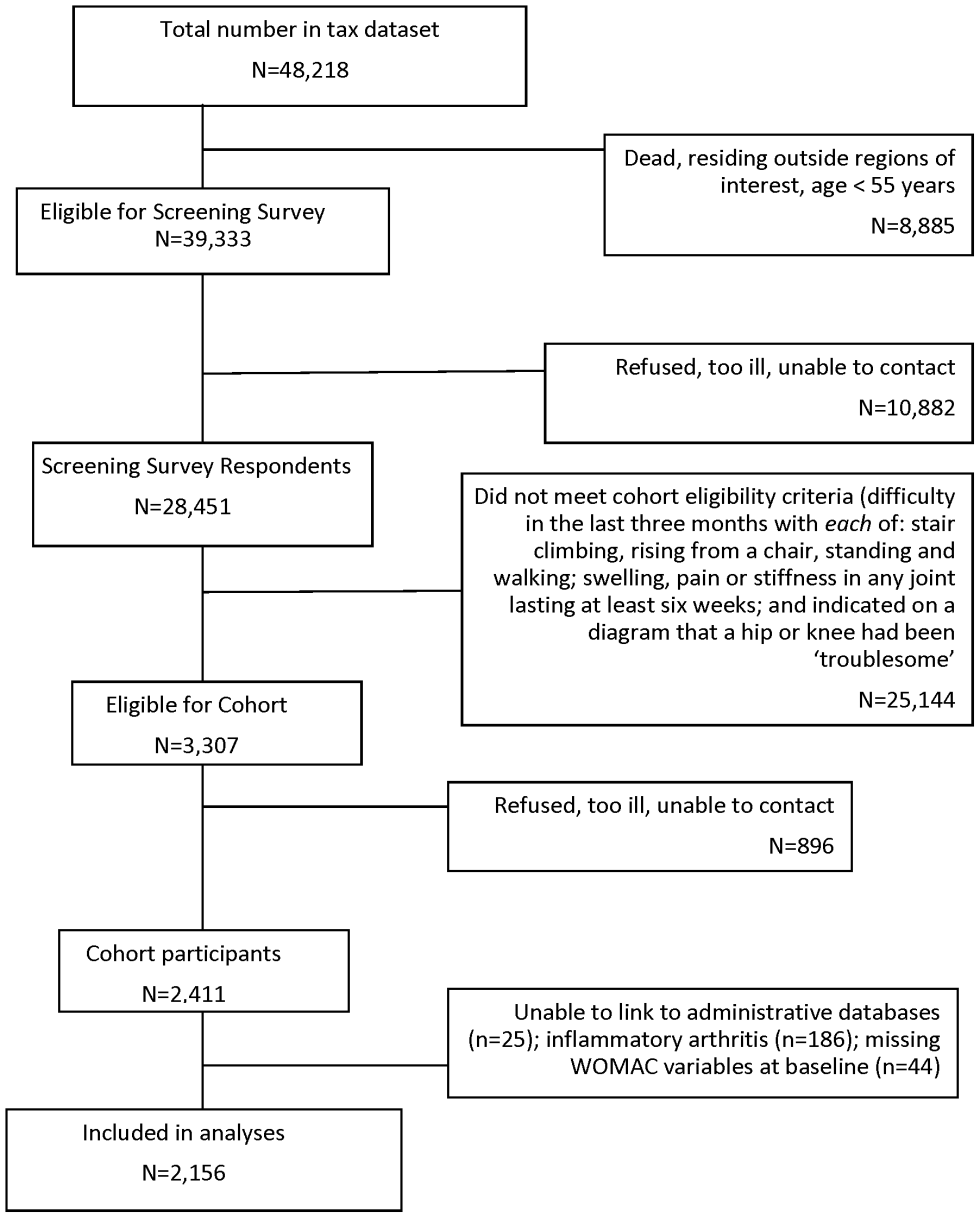
Cohort recruitment flow chart.

The Ontario Hip and Knee Study is a population-based study of people who reported at least moderately severe pain and disability from hip and/or knee OA. The cohort was recruited from 1996–98 through a screening survey of 100% of the population aged 55+ years in two Ontario regions – one rural and one urban – that identified individuals with symptomatic hip/knee arthritis [20]. Of 28,451 screening survey respondents (72.3% response rate), those with: difficulty in the last three months with *each* of stair climbing, rising from a chair, standing and walking; swelling, pain or stiffness in any joint lasting at least six weeks; and who indicated on a diagram that a hip or knee had been ‘troublesome’ were invited to participate. Of 2,411 that agreed, 2,225 had OA **(** **Figure 1** **).** A subsequent validation study found that 96% who met cohort criteria had hip and/or knee arthritis on examination and radiographs.

### Assessments

We used data collected at baseline through a standardized mail/telephone survey linked, with participants’ consent, to provincial health administrative databases: a) physician services from the Ontario Health Insurance Plan (OHIP) Physician and Laboratory Billing Records; b) inpatient hospitalizations and same day surgeries from the Canadian Institute for Health Information (CIHI) hospital discharge abstract database (DAD) and National Ambulatory Care Reporting System (NACRS) database (from April, 2002); c) Emergency Department (ED) visits - prior to April, 2002, extracted from the Physician Billing Records; as of April, 2002, ED visits were obtained from the NACRS database; and prescription drug use from the Ontario Drug Benefits records, for those aged 65+ years and thus eligible for provincial drug benefits.

The baseline survey assessed: socio-demographics; living circumstances; smoking; self-reported height, weight and NSAID use; number of troublesome (painful, aching, stiff or swollen) hips and knees using a joint homunculus; comorbidity (participants indicated if they had been diagnosed by a physician and received treatment in the past year for ‘high blood pressure’, ‘hardening of the arteries’, diabetes, and ‘heart problems’); and mental health status (SF-36 mental health subscale; scores <60/100 indicate probable depression [21]). OA symptom severity was assessed using the reliable and valid Western Ontario McMaster Universities OA Index, WOMAC, pain and physical function subscales [22]. The five-item pain subscale assesses the amount of pain experienced in the hips and knees in the previous week with walking on flat ground, going up/down stairs, at night in bed, sitting or lying, and standing upright. The 17-item physical function subscale assesses the amount of difficulty experienced in the past week performing each of 17 activities ranging from sitting to heavy household chores. Response options are from 0 (none) to 4 (extreme). For each scale, item scores were summed and then normalized to lie between 0 and 100; higher scores indicate worse OA symptoms. Baseline walking disability was additionally assessed using the 2-item Health Assessment Questionnaire [23], HAQ, walking subscale, and use of an aid (e.g., cane or walker) for ambulation (yes/no). The HAQ walking scale assesses level of difficulty in the prior week walking outdoors on flat ground and climbing up five steps, from none (0) to unable to do (3), and use of aids or devices to perform these activities. Higher scores indicate greater difficulty walking.

The presence of hypertension or diabetes at baseline was based on *either* self-reported physician diagnosis ‘ever’ *or* meeting validated criteria for inclusion in provincial disease registries [24], [25]. History of CVD prior to cohort recruitment (pre-existing CVD) was defined as ‘present’ if the participant had experienced ≥1 hospital discharge for acute myocardial infarction (AMI), congestive heart failure (CHF), stroke or transient ischemic attack (TIA), or receipt of coronary revascularization, within the previous five years [26]. Using physician claims, the number of ambulatory visits to a primary care physician and specialists in the pre-baseline year were assessed as proxies for health status. The Johns Hopkins University Adjusted Clinical Group system was used to classify participants as ‘frail’ (yes/no) [27]. Receipt of post-baseline hip or knee joint replacement procedures (elective and non-elective primary and revision TJA) was determined using hospital discharge data; the procedure and diagnostic codes used to identify joint replacement procedures may be found at http://links.lww.com/A1274.

Our primary outcome was all cause-mortality from baseline to February 28, 2012, determined from hospital discharge abstracts and death certificates from the Registered Persons Database. Our secondary outcome was a composite CVD outcome, defined as an ED visit or hospitalization for AMI, coronary revascularization (CABG or PCI), CHF, stroke or TIA.

### Statistical Analysis

Baseline cohort characteristics and the proportions that experienced each outcome were calculated using proportions, means and medians as appropriate. Cox proportional hazards regression was used to examine the contribution of baseline OA symptom severity (WOMAC pain subscale, WOMAC function subscale, HAQ walking score and use of a walking aid, separately) to all-cause mortality after controlling for potential confounders and to examine time to our composite CVD outcome, censoring on death. For each model, we assessed the following baseline covariates: socio-demographics, probable depression, hypertension, diabetes, pre-existing CVD, number of *other* self-reported conditions, body mass index (BMI), smoking, and self-reported NSAID use. Missing baseline values for height, weight or smoking status were imputed using post-baseline information. Age, sex, BMI, smoking status, diabetes, pre-existing CVD, and hypertension were included in all multivariable models; additional covariates were those associated with the outcome of interest at p<0.05. In our final models, we tested for interactions between baseline OA severity (WOMAC scores, HAQ walking score, use of walking aids) and pre-existing CVD.

#### Secondary analyses

Non-ASA NSAIDs have been shown to increase risk for CVD events. Thus, the relationship between worse OA symptoms and occurrence of CVD events or death, if found, may be explained, in part, by use of prescription NSAIDs. In a secondary analysis, we therefore controlled for receipt of a prescription for a non-ASA NSAID in cohort members eligible for drug benefits during the pre-baseline year (age 66+ years at baseline). Finally, we have previously shown that, in the absence of receipt of total joint arthroplasty (TJA), people with hip and knee OA experience worsening of their functional limitations over time [28], [29]; receipt of primary, elective TJA of the hip or knee is associated with significant improvement in OA pain and walking disability. Thus, we also assessed the effect of baseline measures of OA disability and our outcomes of interest after further adjustment for post-baseline receipt of a primary, elective hip or knee TJA, modeled as a time-dependent covariate. We hypothesized that receipt of TJA would be associated with an attenuation of the effect of baseline OA severity on our primary and secondary outcomes.

In all analyses, individuals were censored if they emigrated, died or at the end of available data (February 28, 2012). Analyses were conducted using SAS (Version 9, SAS Institute, Cary, North Carolina).

## Results

### Baseline Cohort Characteristics

Of the 2,225 baseline cohort participants with hip/knee OA, 25 could not be linked with administrative data and 44 were missing baseline WOMAC function scores, leaving 2,156 individuals for our analyses **(** **Figure 1** **)**. Their mean age was 71.3 years (SD 9.2) and 72.0% were female; 39.6% had pre-existing CVD, 20.4% had diabetes and 63.5% hypertension, 28.8% met criteria for probable depression, 33.7% were obese and 37.2% and 14.8% were past and current smokers, respectively. Mean baseline WOMAC pain and function scores (/100) were 40.9 (SD 21.9) and 40.6 (SD 20.8), respectively. Forty-four percent reported using walking aids; 37.9% reported NSAID use. **Table 1** **.**

**Table 1:** Sample characteristics by baseline level of physical function (WOMAC Function Subscale Quartiles).

		WOMAC Function Quartile				
Participant Characteristic	Overall(N = 2156)	1^st^ (leastaffected)(N = 502)	2^nd^ (N = 530)	3^rd^ (N = 557)	4^th^ (mostaffected)(N = 567)	P-value*
**Demographics** **(Baseline Assessment)**						
Age: mean (SD)	71.3 (9.2)	70.4 (8.9)	70.4 (8.7)	72.0 (9.0)	72.1 (10.1)	<0.001
Age category: N (%)						<0.001
55–64	591 (27.4%)	150 (29.9%)	148 (27.9%)	133 (23.9%)	160 (28.2%)	
65–74	767 (35.6%)	192 (38.3%)	220 (41.5%)	194 (34.8%)	161 (28.4%)	
75+	798 (37.0%)	160 (31.9%)	162 (30.6%)	230 (41.3%)	246 (43.4%)	
Sex: N (%) female	1,552 (72.0%)	348 (69.3%)	366 (69.1%)	401 (72.0%)	437 (77.1%)	0.01
Income: N (%)						<0.001
≤ $20,000	1,132 (52.5%)	220 (43.8%)	251 (47.4%)	293 (52.6%)	368 (64.9%)	
> $20,000	649 (30.1%)	198 (39.4%)	187 (35.3%)	163 (29.3%)	101 (17.8%)	
Missing	375 (17.4%)	84 (16.7%)	92 (17.4%)	101 (18.1%)	98 (17.3%	
Education: N (%)						<0.001
Less than high school completion	778 (36.1%)	145 (28.9%)	161 (30.4%)	221 (39.7%)	251 (44.3%)	
Completed high school	1,013 (47.0%)	247 (49.2%)	263 (49.6%)	266 (47.8%)	237 (41.8%)	
Some post-secondary education	327 (15.2%)	105 (20.9%)	100 (18.9%)	60 (10.8%)	62 (10.9%)	
Missing	38 (1.8%)	5 (1.0%)	6 (1.1%)	10 (1.8%)	17 (3.0%)	
Living arrangements: N (%)						0.02
Living with others	1,425 (66.1%)	350 (69.7%)	374 (70.6%)	360 (64.6%)	341 (60.1%)	
Living alone	671 (31.1%)	138 (27.5%)	145 (27.4%)	183 (32.9%)	205 (36.2%)	
In long-term care	33 (1.5%)	9 (1.8%)	5 (0.9%)	7 (1.3%)	12 (2.1%)	
Missing	27 (1.3%)	5 (1.0%)	6 (1.1%)	7 (1.3%)	9 (1.6%)	
Place of residence: N (%) urban	942 (43.7%)	271 (44.0%)	225 (42.5%)	225 (40.4%)	271 (47.8%)	0.08
**Health-Related Variables** **(Baseline Assessment)**						
Pre-existing CVD (self-reported ordocumented in the administrative data): N (%)	854 (39.6%)	159 (31.7%)	188 (35.5%)	233 (41.8%)	274 (48.3%)	<0.001
Diabetes (self reported or documentedin the administrative data): N (%)	440 (20.4%)	87 (17.3%)	95 (17.9%)	114 (20.5%)	144 (25.4%)	0.003
Hypertension (self reported ordocumented in the administrative data):N (%)	1,369 (63.5%)	302 (60.2%)	323 (60.9%)	366 (65.7%)	378 (66.7%)	0.06
Depression (SF36 mental health subscale):N (%)	621 (28.8%)	83 (16.5%)	123 (23.2%)	154 (27.7%)	261 (46.0%)	<0.001
No. other comorbid conditions (self reported):N (%)						<0.001
0	84 (3.9%)	37 (7.4%)	19 (3.6%)	12 (2.2%)	16 (2.8%)	
1	390 (18.1%)	128 (25.5%)	101 (19.1%)	105 (18.9%)	56 (9.9%)	
2	678 (31.5%)	176 (35.1%)	178 (33.6%)	186 (33.4%)	138 (24.3%)	
3+	1,004 (46.6%)	161 (32.1%)	232 (43.8%)	254 (45.6%)	357 (63.0%)	
Frailty: N (%)	54 (2.5%)	12 (2.4%)	7 (1.3%)	16 (2.9%)	19 (3.4%)	0.17
Body mass index (BMI): N (%)						0.007
Underweight	36 (1.7%)	5 (1.0%)	6 (1.1%)	12 (2.2%)	13 (2.3%)	
Normal	546 (25.4%)	152 (30.3%)	127 (24.0%)	148 (26.6%)	119 (21.0%)	
Overweight	761 (35.3%)	181 (36.1%)	200 (37.7%)	188 (33.8%)	192 (33.9%)	
Obese	683 (31.7%)	136 (27.1%)	174 (32.8%)	170 (30.5%)	203 (35.8%)	
Missing	130 (6.0%)	28 (5.6%)	23 (4.3%)	39 (7.0%)	40 (7.1%)	
Smoking history: N (%)						0.55
Never smoked	1,012 (46.9%)	231 (46.0%)	239 (45.1%)	270 (48.5%)	272 (48.0%)	
Former smoker	783 (36.3%)	194 (38.7%)	197 (37.2%)	205 (36.8%)	187 (33.0%)	
Current smoker	312 (14.5%)	67 (13.4%)	81 (15.3%)	72 (12.9%)	92 (16.2%)	
Missing	49 (2.3%)	10 (2.0%)	13 (2.5%)	10 (1.8%)	16 (2.8%)	
Number of visits to a general practitioner inpre-baseline year: median (inter-quartilerange)	6 (3–11)	6 (2–10)	5 (3–10)	6 (3–11)	8 (4–12)	<0.001
Number of visits to a specialist in pre-baselineyear: median (inter-quartile range)	2 (1–6)	2 (1–6)	2 (0–5)	2 (1–6)	3 (1–7)	0.005
Continuity of Care Index (range is from 0 to 1)	0.90 (0.74–1.0)	0.90 (0.71–1.0)	0.93 (0.76–1.0)	0.90 (0.72–1.0)	0.89 (0.74–1.0)	0.03
**Osteoarthritis-Related Variables**						
Use of aid for walking: N (%)	953 (44.2%)	130 (25.9%)	179 (33.8%)	267 (47.9%)	377 (66.5%)	<0.001
HAQ walking score (/3): median (IQR)	2 (0–2)	0 (0–2)	1 (0–2)	2 (1–2)	2 (2–2)	<0.0001
WOMAC pain quartile: N (%)						<0.001
1^st^ (least pain)	460 (21.3%)	317 (63.2%)	97 (18.3%)	33 (5.9%)	13 (2.3%)	
2^nd^	435 (20.2%)	116 (23.1%)	210 (39.6%)	92 (16.5%)	17 (3.0%)	
3^rd^	627 (29.2%)	59 (11.8%)	172 (32.5%)	262 (47.0%)	134 (23.6%)	
4^th^ (most pain)	627 (29.2%)	7 (1.4%)	49 (9.3%)	169 (30.3%)	402 (70.9%)	
Missing	7 (0.3%)	3 (0.6%)	2 (0.4%)	1 (0.2%)	1 (0.2%)	
Non-ASA NSAID use (self-report): N (%)	816 (37.9%)	163 (32.5%)	222 (41.9%)	211 (37.9%)	220 (38.8%)	0.02
Number of troublesome hips/knees: N (%)						<0.001
0	84 (3.9%)	44 (8.8%)	21 (4.0%)	9 (1.6%)	10 (1.8%)	
1	310 (14.4%)	101 (20.1%)	94 (17.7%)	77 (13.8%)	38 (6.7%)	
2	786 (36.5%)	179 (35.7%)	199 (37.6%)	213 (38.2%)	195 (34.4%)	
3	249 (11.6%)	39 (7.8%)	59 (11.1%)	75 (13.5%)	76 (13.4%)	
4	454 (21.1%)	35 (7.0%)	103 (19.4%)	109 (19.6%)	207 (36.5%)	
Missing	273 (12.7%)	104 (20.7%)	54 (10.2%)	74 (13.3%)	41 (7.2%)	
**Follow-up Time and Outcomes**						
Follow-up time until death or censoring (years):median (inter-quartile range)	13.2 (6.3–15.5)	13.9 (7.5–15.5)	13.9 (7.8–15.6)	13.0 (5.8–15.5)	10.6 (5.0–15.3)	<0.001
Outcome of death: N (%)	1,236 (57.3%)	269 (21.8%)	265 (21.4%)	331 (26.8%)	371 (30.0%)	<0.001
Follow-up time until composite CVDoutcome or censoring): median (inter-quartilerange)	9.2 (3.7–15.0)	10.5 (4.8–15.2)	10.6 (4.7–15.4)	8.8 (3.3–14.9)	6.9 (3.1–14.6)	<0.001
CVD-related hospital admission orED visit: N (%)	822 (38.1%)	182 (36.3%)	189 (35.7%)	213 (38.2%)	238 (42.0%)	0.13

CVD = Cardiovascular disease.

*P-values test the hypothesis that of no differences among the four WOMAC function quartiles and were obtained using ANOVA (continuous, normally distributed covariates), Wilcoxon rank sum tests (ordinal covariates), and chi-square tests (categorical covariates).

### Effect of Baseline OA Pain and Disability on All-Cause Mortality

**Figure 2:**
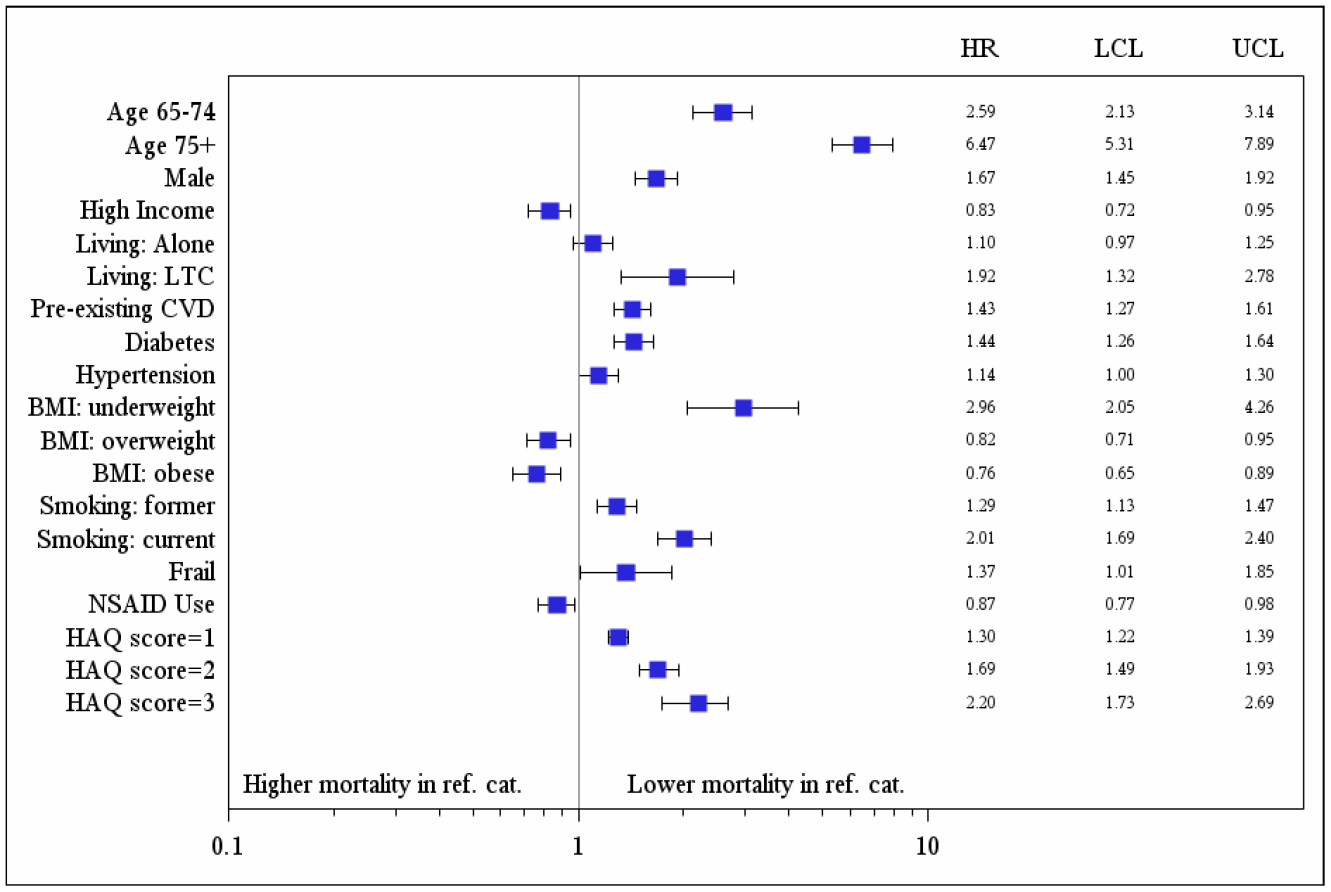
Results of multivariable analysis: independent predictors of all-cause mortality.

Over a median 13.2 years of follow-up (inter-quartile range, IQR, 6.3–15.5 years), 1,236 (57.3%) participants died. Measures of baseline OA-related disability, but not pain, were associated with increased risk for all cause death in unadjusted analyses (Hazard Ratio, HR, per 10-point increase in WOMAC function score 1.08, 95% Confidence Interval, CI, 1.05–1.11, p<0.001; HR per unit increase in HAQ walking score 1.63, 95% CI 1.53–1.74, p<0.001; HR for use of walking aids 2.28, 95% CI 2.04–2.55, p<0.001; HR per 10-point increase in WOMAC pain score 1.00, 95% CI 0.98–1.03, p = 0.85). Additional predictors of all cause mortality are shown in **Table 2**. Other predictors of higher mortality in the multivariable model were older age, male sex, lower income, nursing home residence, pre-existing CVD, diabetes, hypertension, being underweight, smoking and frailty; self-reported NSAID use and being overweight or obese were protective. Controlling for these factors, all-cause mortality was independently predicted by higher (worse) baseline WOMAC function scores (adjusted HR per 10-point increase in score 1.04, 95% CI 1.01–1.07, p = 0.004) and by walking disability (adjusted HR per unit increase in the HAQ walking score 1.30, 95% CI 1.22–1.39, p<0.001; use of a walking aid 1.51, 95% CI 1.34–1.70, p<0.0001). Model fit was best with the HAQ walking score (explained variance 0.57) [30] **(** **Figures 2** **and** **3** **).** There were no significant interactions between pre-existing CVD and baseline OA disability (HAQ walking score, p  = 0.31; use of an aid, p = 0.64; WOMAC function, p = 0.70). However, there was a significant interaction between pre-existing CVD and baseline WOMAC pain score (p = 0.009); greater baseline OA pain predicted higher mortality in those with, but not without, pre-existing CVD (adjusted HR per 10-point increase in WOMAC pain score  = 1.04, 95% CI 1.00–1.08, p  = 0.038 and 0.97, 95% CI 0.93–1.01, p  = 0.96, respectively).

**Figure 3:**
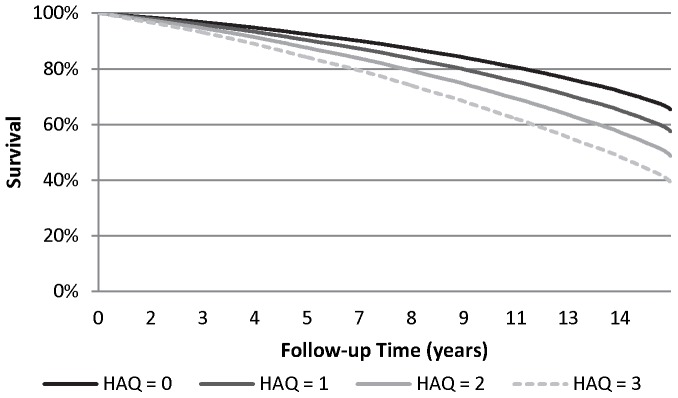
Survival curves for the typical osteoarthritis (OA) cohort participant* by level of walking disability (HAQ walking score) at baseline assessment. *typical defined as the mean or most common value for variables in the final model for all-cause death.

**Table 2:** Sample Characteristics at Baseline by Occurrence of All-Cause Death (values are numbers and percentages unless otherwise stated).

Predictor Variables	Participant Died		Crude Hazard Ratio*(95%confidence interval)	p-value*
	No (N = 920)	Yes (N = 1236)		
Age:				<0.001
55–64 (reference)	445 (48.4%)	146 (11.8%)	1.0	
65–74	365 (39.7%)	402 (32.5%)	2.60 (2.15–3.14)	
75+	110 (12.0%)	688 (55.7%)	7.11 (5.94–8.52)	
Male sex	215 (23.4%)	389 (31.5%)	1.34 (1.19–1.52)	<0.001
Income:				<0.001
Less than $20K (reference)	440 (47.8%)	692 (56.0%)	1.0	
Greater than $20K	324 (35.2%)	325 (26.3%)	0.73 (0.64–0.84)	<0.001
Missing	156 (17.0%)	219 (17.7%)	0.92 (0.79–1.07)	0.28
Education:				<0.001
Less than high school (reference)	294 (32.0%)	484 (39.2%)	1.0	
High school graduation	451 (49.0%)	562 (45.5%)	0.81 (0.72–0.92)	<0.001
Post-secondary education	165 (17.9%)	162 (13.1%)	0.70 (0.58–0.83)	<0.001
Missing	10 (1.1%)	28 (2.3%)	1.29 (0.88–1.89)	0.19
Living arrangements:				<0.001
Lives with others (reference)	691 (75.1%)	734 (59.4%)	1.0	
Lives alone	220 (23.9%)	451 (36.5%)	1.54 (1.37–1.73)	<0.001
Living in long term care residence	2 (0.2%)	31 (2.5%)	4.42 (3.08–6.34)	<0.001
Missing	7 (0.8%)	20 (1.6%)	1.89 (1.21–2.95)	0.005
Urban place of residence	386 (42.0%)	556 (45.0%)	1.07 (0.96–1.20)	0.21
Pre-existing CVD risk	228 (24.8%)	626 (50.7%)	2.11 (1.89–2.36)	<0.001
Diabetes	126 (13.7%)	314 (25.4%)	1.64 (1.44–1.86)	<0.001
Hypertension	506 (55.0%)	863 (69.8%)	1.54 (1.37–1.74)	<0.001
Depression(SF36-mental health subscale)	243 (26.4%)	378 (30.6%)	1.18 (1.05–1.33)	0.007
No. other comorbid conditions:			1.11 (1.04–1.18)	0.003
0	37 (4.0%)	47 (3.8%)		
1	176 (19.1%)	214 (17.3%)		
2	314 (34.1%)	364 (29.5%)		
3+	393 (42.7%)	611 (49.4%)		
Frailty	6 (0.7%)	48 (3.9%)	2.67 (2.00–3.56)	<0.001
Body mass index (BMI):				<0.001
Underweight	3 (0.3%)	33 (2.7%)	3.23 (2.26–4.63)	<0.001
Normal (reference)	208 (22.6%)	338 (27.4%)	1.0	
Overweight	322 (365.0%)	439 (35.5%)	0.84 (0.72–0.96)	0.013
Obese	361 (39.2%)	322 (26.1%)	0.54 (0.54–0.73)	<0.001
Missing	26 (2.8%)	104 (8.4%)	1.73 (1.39–2.16)	<0.001
Smoking history:				0.049
Never smoked (reference)	453 (49.2%)	559 (45.2%)	1.0	
Former smoker	331 (36.0%)	452 (36.6%)	1.08 (0.95–1.22)	0.23
Current smoker	119 (12.9%)	193 (15.6%)	1.24 (1.05–1.46)	0.010
Missing	17 (1.9%)	32 (2.6%)	1.29 (0.90–1.84)	0.16
No. visits to a primary carephysician in pre-baseline year:median (IQR)†	6 (3–10)	7 (4–12)	1.13 (1.08–1.19)	<0.001
No. visits to a specialist inpre-baseline year: median (IQR)†	2 (0–5)	3 (1–7)	1.12 (1.08–1.17)	<0.001
WOMAC Scores: Mean (SD)‡				
Physical function/100	38.1 (20.0)	42.4 (21.2)	1.08 (1.05–1.11)	<0.001
Pain/100	41.0 (20.9)	40.8 (22.5)	1.00 (0.98–1.03)	0.85
HAQ walking score (/3): mean (IQR)	1 (0–2)	2 (1–2)	1.63 (1.53–1.74)	<0.001
Uses an aid to walk	953 (44.2%)	692 (56.0%)	2.28 (2.04–2.55)	<0.001
Self-reported NSAID use	357 (38.8%)	459 (37.1%)	0.90 (0.80–1.01)	0.080

*Univariable hazard ratios, 95% confidence intervals and P values were derived from Cox regression models; missing baseline data was either imputed from responses on the year 1 follow-up assessment or incorporated as a ‘missing’ dummy variable. Hazard ratios >1 indicate lower mortality in reference category.

† Hazard ratios are per doubling of the number of visits in the pre-baseline year.

‡ Hazard ratios are per 10-point increase in WOMAC subscale score.

### Effect of Baseline OA Pain and Disability on Time to Composite CVD Outcome

Over a median 9.2 years of follow-up (IQR 3.7–15.0 years), 822 (38.1%) experienced one or more CVD events (193 AMI, 292 CHF, 288 TIA/stroke, and 71 CABG/PCI). Censoring on death, time to occurrence of the first CVD event was reduced in those with greater baseline OA disability (crude HR per 10-point increase in WOMAC function score  = 1.10, 95% CI 1.05–1.16, p<0.001; crude HR per unit increase in HAQ walking score  = 1.40; 95% CI 1.30–1.51, p<0.001; crude HR for use of walking aids 1.75, 95% CI 1.53–2.01, p<0.001), but unrelated to baseline OA pain (crude HR per 10-point increase in WOMAC pain score  = 1.08; 95% CI 0.92–1.07, p = 0.34). Additional significant predictors are shown in **Table 3** **.** In our final model, risk of a CVD outcome was significantly associated with older age, male sex, pre-existing CVD, diabetes, hypertension, smoking, and with walking disability (adjusted HR per unit increase in HAQ walking score  = 1.17, 95% CI 1.08–1.27, p<0.001; adjusted HR for baseline use of a walking aid  = 1.28, 95% CI 1.11–1.48, p<0.001) **(** **Table 4** **).** We found no significant interactions between baseline OA symptom severity measures and pre-existing CVD (data not shown).

**Table 3:** Sample characteristics at baseline by occurrence of a subsequent composite cardiovascular outcome**.**

Predictor Variables	Participant Experienced CompositeCVD Outcome		Crude Hazard Ratio*(95% confidence interval)	p-value
	No (N = 1,334)	Yes (N = 822)		
Age category: N (%)				<0.001
55–64 (reference)	447 (33.5%)	144 (17.5%)	1.0	
65–74	457 (34.3%)	310 (37.7%)	2.07 (1.70–2.52)	<0.001
75+	430 (32.2%)	368 (44.8%)	3.55 (2.92–4.32)	<0.001
Male sex: N (%)	360 (27.0%)	244 (29.7%)	1.27 (1.09–1.48)	0.002
Income : N (%)				0.0036
Less than $20K (reference)	673 (50.5%)	459 (55.8%)	1.0	
Greater than $20K	430 (32.2%0	219 (26.6%)	0.76 (0.65–0.89)	0.0008
Missing	231 (17.3%)	144 (17.5%)	0.90 (0.74–1.08)	0.90
Education: N (%)				0.001
Less than high school (reference)	458 (34.3%)	320 (38.9%)	1.0	
High school graduation	641 (48.1%)	372 (45.3%)	0.82 (0.71–0.95)	0.009
Post-secondary education	217 (16.3%)	110 (13.4%)	0.73 (0.59–0.90)	0.004
Missing	18 (1.4%)	20 (2.4%)	1.41 (0.90–2.21)	0.14
Living arrangements: N (%)				<0.001
Lives with others (reference)	897 (67.2%)	528 (64.2%)	1.0	
Lives alone	401 (30.1%)	270 (32.9%)	1.24 (1.07–1.43)	0.005
Long term care residence	19 (1.4%)	14 (1.7%)	2.53 (1.48–4.30)	<0.001
Missing	17 (1.3%)	10 (1.2%)	1.18 (0.63–2.21)	0.60
Urban place of residence: N (%)	585 (43.9%)	357 (43.4%)	1.01 (0.88–1.16)	0.91
Pre-existing CVD: N (%)	425 (31.9%)	429 (52.2%)	2.42 (2.11–2.77)	<0.001
Diabetes: N (%)	209 (15.7%)	231 (28.1%)	2.01 (1.72–2.34)	<0.001
Hypertension: N (%)	786 (58.9%)	583 (70.9%)	1.68 (1.44–1.95)	<0.001
Depression(SF36-mental health subscale) : N (%)	380 (28.5%)	241 (29.3%)	1.11 (0.95–1.28)	0.19
No. other comorbid conditions†			1.07 (1.01–1.14)	0.025
0	46 (3.5%)	38 (4.6%)		
1	257 (19.3%)	133 (16.2%)		
2	426 (31.9%)	252 (30.7%)		
3+	605 (45.4%)	399 (48.5%)		
Frailty: N (%)	30 (2.3%)	24 (2.9%)	1.85 (1.23–2.77)	0.003
Body mass index (BMI): N (%)				<0.001
Underweight	26 (2.0%)	10 (1.2%)	1.37 (0.89–1.28)	0.33
Normal (reference)	359 (26.9%)	187 (22.8%)	1.0	
Overweight	465 (34.9%)	296 (36.0%)	1.06 (0.89–1.28)	0.50
Obese	415 (31.1%)	268 (32.6%)	1.02 (0.85–1.23)	0.85
Missing	69 (5.2%)	61 (7.4%)	1.85 (1.39–2.48)	<0.001
Smoking history: N (%)				0.19
Never smoked (reference)	629 (47.2%)	383 (46.6%)	1.0	
Former smoker	481 (36.1%)	302 (36.7%)	1.07 (0.92–1.24)	0.42
Current smoker	200 (15.0%)	112 (5.2%)	1.01 (0.82–1.24)	0.95
Missing	24 (1.8%)	25 (3.0%)	1.55 (1.03–2.32)	0.035
No. visits to a primary care physician inpre-baseline year: median (IQR)‡	6 (3–11)	7 (4–11)	1.18 (1.11–1.25)	<0.001
No. visits to a specialist inpre-baseline year: median (IQR)‡	2 (0–6)	3 (1–6)	1.10 (1.05–1.15)	<0.001
WOMAC Scores: Mean (SD)§				
Physical function/100	39.8 (20.5)	41.7 (21.2)	1.10 (1.05–1.16)	<0.001
Pain/100	40.7 (21.5)	41.2 (22.5)	1.08 (0.92–1.27)	0.34
HAQ walking score/3: median (IQR)	1 (0–2)	2 (1–2)	1.40 (1.30–1.51)	<0.001
Use of aid for walking: N (%)	542 (40.6%)	411 (50.0%)	1.75 (1.53–2.01)	<0.001
Self-reported NSAID use: N (%)	494 (37.0%)	322 (39.2%)	1.03 (0.90–1.19)	0.65
Number of troublesome hips/knees: N (%)			0.95 (0.90–1.00)	0.050
0	212 (15.9%)	145 (17.6%)		
1	197 (14.8%)	113 (13.8%)		
2	493 (37.0%)	293 (35.6%)		
3	139 910.4%)	110 (13.4%)		
4	293 (22.0%)	161 (19.6%)		

*Univariable hazard ratios, 95% confidence intervals and P values were derived from a survival model, censoring on death; missing baseline data were either imputed from responses on the year 1 follow-up assessment or incorporated as a ‘missing’ dummy variable. Hazard ratios >1 indicate lower mortality in reference category.

† Hazard ratio is per additional comorbid condition.

‡ Hazard ratios are per doubling of the number of visits in the pre-baseline year.

§ Hazard ratios are per 10-point increase in WOMAC subscale score.

**Table 4:** Results of multivariable survival analysis: effect of baseline osteoarthritis (OA) pain and disability on risk for composite cardiovascular (CVD) outcome after adjusting for covariates and censoring on death.

Participant Characteristic	Adjusted Hazard Ratio	95% confidence interval	p-value
**Age:**			<0.001
55–64 (reference)	1.0		
65–74	1.90	1.55–2.33	<0.001
75+	3.05	2.46–3.78	<0.001
**Male** (female is reference)	1.31	1.11–1.54	0.001
**Pre-existing cardiovascular disease**	1.86	1.61–2.15	<0.001
**Diabetes**	1.67	1.43–1.96	<0.001
**Hypertension**	1.20	1.02–1.40	0.026
**Body mass index (BMI):**			0.20
Underweight	1.36	0.72–2.58	0.35
Normal (reference)	1.0		
Overweight	1.07	0.89–1.29	0.49
Obese	1.11	0.92–1.35	0.28
Missing	1.42	1.06–1.92	0.021
**Smoking history:**			0.016
Never smoked (reference)	1.0		
Former smoker	1.16	0.98–1.36	0.080
Current smoker	1.38	1.10–1.72	0.004
Missing	1.47	0.95–2.27	0.083
**HAQ walking score** (per unit increase in score)	1.17	1.08–1.27	<0.001

Model R-square  = 0.381.

### Secondary Analyses

When we restricted our analyses to the 1,501 participants who were ≥66 years at baseline, and thus had been eligible for drug benefits for at least one year, and further adjusted for receipt of a prescription for a non-ASA NSAID, our results were unchanged (data not shown).

Among the 2,156 OA cohort participants, 402 (18.65%) received a post-baseline primary, elective hip or knee TJA prior to death or censoring. Of these, most (93.8%) received their TJA prior to experiencing a CVD event or censoring. Additional adjustment of our final models for receipt of a post-baseline primary, elective TJA found that TJA was protective of both our primary and secondary outcomes (adjusted HR for all-cause death  = 0.62, 95% CI 0.51–0.76, p<0.001; adjusted HR for CVD events  = 0.66, 95% CI 0.52–0.85, p<0.001), but did not affect the adjusted HRs for baseline measures of OA pain or disability (data not shown).

## Discussion

This population-based cohort study of persons with hip and knee OA followed for over a decade found a significant association between greater OA-related disability and both all-cause mortality and risk for a serious CVD event. Survival was also reduced among those with pre-existing CVD who had greater baseline OA pain. Our findings are important. As the proportion of the population that is affected by multiple chronic conditions increases, it is critical to understand the impact of comorbidity on health outcomes. Among the most common disease dyads is osteoarthritis and ischemic heart disease, estimated to affect one in five Medicare beneficiaries [12]. Our findings therefore have substantial implications for clinical practice and quality improvement to optimize outcomes in people with OA and heart disease.

Our results confirm those of Nuesch *et al* [19], who documented increased all-cause mortality in individuals with hip and knee OA relative to the age- and sex-matched population of England, and linked increased mortality risk with walking disability. Additionally, our results extend their work in showing that *within individuals with hip and knee OA,* severity of OA-related pain and disability are significant predictors of future risk for all-cause death. These effects remained after controlling for multiple potential confounders, including obesity and obesity-related conditions, social health determinants and mental health status. Of the OA severity measures assessed, walking disability assessed at baseline using the two-item HAQ walking scale had the greatest ability to predict survival such that survival at the end of our follow-up period was approximately 25% lower among those with the highest (worse) versus lowest scores for walking disability at baseline. Further research is warranted to elucidate the mechanisms by which reduced mobility due to OA impacts survival.

The prior study by Nuesch *et al* found that increased mortality in people with OA was largely due to CVD causes. As noted above, OA-related disability may increase cardiac risk by limiting ability to engage in physical activity and decreasing cardio-respiratory fitness [16], [31], [32]. Thus, we tested for an independent effect of baseline OA disability on risk for serious CVD events. Controlling for multiple potential confounders, we found that risk for our composite CVD outcome was almost 30% higher among those with versus without self-reported use of walking aids. Further research is warranted to elucidate the extent to which increased risk for CVD events due to walking disability contributes to reduced survival in people with hip and knee OA. We also observed a positive significant relationship between baseline OA pain severity and all-cause death among those with pre-existing CVD; this finding is intriguing and warrants further study.

Two-thirds of our OA participants were overweight or obese, two-thirds had hypertension, almost forty percent had pre-existing CVD, and one-fifth had diabetes. In such individuals, functional limitations due to OA may impact ability to participate in physical activity, contributing to weight gain and physical deconditioning, worse disease control, and increased risk for adverse outcomes including death. Although, to date, no study has explicitly evaluated the contribution of OA to the management and outcomes of other common chronic conditions, there is indirect support for a relationship. In a survey of three groups of mainly male US Veterans aged 66–74 years (heart failure; diabetes; and general primary care users), activity limitations due to chronic pain were commonly reported (60% in all groups). Most reported back, hip or knee pain [33]. Of the 60% who reported pain limiting activities, most were managing their pain with rest or sedentary activities (e.g. watching TV) and fewer than half were exercising. Collectively, these findings underscore the need to move away from single-disease focused clinical practice guidelines towards chronic disease strategies that consider the potential for common approaches to chronic disease prevention and management, including targeted interventions to address mobility.

Total joint arthroplasty (TJA) of the hip and knee is a highly effective treatment for advanced hip and knee OA[34]–[36]. Thus, if walking disability increases risk for CVD events or death, receipt of a TJA procedure may be protective. Indeed, in a secondary analysis, we found that post-baseline receipt of a primary, elective hip or knee TJA was associated with improved survival and reduced risk for CVD events, but did not affect the observed relationships between measures of OA disability and our outcomes. Controlling for other factors, those who received a post-baseline primary, elective TJA were approximately one third less likely to experience our outcomes. While these findings are intriguing, we cannot rule out a ‘healthy user effect’ as the explanation for these results. In subsequent research using our cohort data, we therefore examined the relationship between receipt of hip or TJA and risk for CVD events using a propensity score matched landmark analysis [37]. Using this approach, TJA remained significantly and independently protective of risk for CVD events. Further research is warranted to elucidate explanations for this relationship, including the role of physical activity, pain relief, and reduction in use of NSAIDs.

Strengths of our study include reliance on an established, well-characterized population-based cohort with hip and knee OA, long duration of follow-up, ascertainment of survival status of all subjects regardless of post-baseline survey participation, and validated outcomes using linked health administrative data. However, there are also some study limitations. First, we were unable to control for lipid levels, abdominal obesity and family history of CVD and we did not consider the effect of medication use to manage CVD risk, such as anti-hypertensive and lipid lowering drugs. Thus, lack of control for unmeasured confounders remains a possible explanation for our findings. Second, we did not control for changes in OA symptom severity – or other covariates, such as CVD status - over the follow-up period as we were interested in the prognostic value of measures of OA disability on our outcomes of interest and participation attrition over time would have reduced our sample size and thus power. This approach may have resulted in misclassification bias. We have previously shown that, in the absence of TJA, OA disability, as measured using the WOMAC, worsens over time [28]. Thus, we may have underestimated the estimated effect of OA symptom severity on our outcomes of interest.

In conclusion, controlling for other factors, our study identified hip/knee OA functional limitations – and specifically walking disability - as a potentially modifiable risk factor for serious CVD events and death. Pragmatic clinical trials of interventions to reduce walking disability in individuals with multi-morbidity, including OA, are needed. Chronic disease strategies that target combinations of clustered conditions – and recognize the impact of OA - are likely to be more effective and are needed.
